# High mortality of blue, humpback and fin whales from modeling of vessel collisions on the U.S. West Coast suggests population impacts and insufficient protection

**DOI:** 10.1371/journal.pone.0183052

**Published:** 2017-08-21

**Authors:** R. Cotton Rockwood, John Calambokidis, Jaime Jahncke

**Affiliations:** 1 Point Blue Conservation Science, Petaluma, California, United States of America; 2 Cascadia Research Collective, Olympia, Washington, United States of America; Institute of Deep-sea Science and Engineering, Chinese Academy of Sciences, CHINA

## Abstract

Mortality from collisions with vessels is one of the main human causes of death for large whales. Ship strikes are rarely witnessed and the distribution of strike risk and estimates of mortality remain uncertain at best. We estimated ship strike mortality for blue humpback and fin whales in U.S. West Coast waters using a novel application of a naval encounter model. Mortality estimates from the model were far higher than current minimum estimates derived from stranding records and are closer to extrapolations adjusted for detection probabilities of dead whales. Our most conservative model estimated mortality to be 7.8x, 2.0x and 2.7x the U.S. recommended limit for blue, humpback and fin whales, respectively, suggesting that death from vessel collisions may be a significant impediment to population growth and recovery. Comparing across the study area, the majority of strike mortality occurs in waters off California, from Bodega Bay south and tends to be concentrated in a band approximately 24 Nm (44.5 km) offshore and in designated shipping lanes leading to and from major ports. While some mortality risk exists across nearly all West Coast waters, 74%, 82% and 65% of blue, humpback and fin whale mortality, respectively, occurs in just 10% of the study area, suggesting conservation efforts can be very effective if focused in these waters. Risk is highest in the shipping lanes off San Francisco and Long Beach, but only a fraction of total estimated mortality occurs in these proportionally small areas, making any conservation efforts exclusively within these areas insufficient to address overall strike mortality. We recommend combining shipping lane modifications and re-locations, ship speed reductions and creation of ‘Areas to be Avoided’ by vessels in ecologically important locations to address this significant source of whale mortality.

## Introduction

Widespread hunting of whales during the nineteen and twentieth centuries has left many whale populations severely depleted [1]. In the eastern North Pacific commercial whaling for humpback and blue whales continued through 1965 when international restrictions to protect these species were implemented, though some illegal hunting continued after that [2]. An international moratorium on whaling implemented in 1985 stopped many declines and led to increases in some whale stocks [3]. In the U.S., marine mammals have legal protection under the Marine Mammal Protection Act (MMPA), making the take of marine mammals illegal. Nonetheless, human-caused mortality of whales still occurs in U.S. waters, in some cases threatening the recovery of depleted populations [4]. One of the most significant human effects on whales is collisions with vessels, which have been identified as a significant source of human-caused mortality for whale populations in the U.S. and around the world [5–8].

Vessel collisions, also known as ship strikes, are relatively rare occurrences with low probability of detection, yet the resulting mortalities are problematic for long-lived, low fecundity whale populations. This makes effective assessment of ship strike mortality both important and exceedingly difficult. On the U.S. East Coast, there have been significant regulatory efforts to decrease mortality from ship collisions with the severely depleted and endangered North Atlantic right whale. Methods have included the re-location of shipping lanes, establishment of ecologically important areas and mandates for ship speed reductions [9]. Additional work has helped justify these regulations by establishing statistical relationships between ship speed and collision risk [10], assessing the probability of mortality when a strike occurs [5,11] and improving our understanding of behavioral responses that may allow whales to avoid impending collisions [12,13]. The mitigation of ship strikes along the East Coast has significantly benefitted right whales, leading to increases in the population over the last decade [7,14].

On the West Coast, strikes of blue (*Balaenoptera musculus*), humpback (*Megaptera novaeangliae*) and fin (*Balaenoptera physalus*) whales are major causes of death for those species, especially the endangered fin and blue whales [15]. Blue, humpback and fin whales migrate seasonally along the West Coast of the U.S., where they overlap with significant shipping activity [6,16–19]. Important feeding hotspots for blue and humpback whales occur in waters near the ports of Long Beach/Los Angeles and Oakland where they intersect with vessel traffic lanes, also known as Traffic Separation Schemes (TSSs) [20–22]. In both these areas, relocation of the lanes was implemented in 2013 and speed reductions are being considered or tested. In southern California, a monetary speed reduction incentive program was implemented in 2014, while off of San Francisco, the National Oceanographic and Atmospheric Administration National Marine Fisheries Service (NOAA NMFS) is currently testing a voluntary ship speed reduction during the peak whale months from July to September.

The Eastern N. Pacific stock of blue whales ranges from the Gulf of Alaska to the eastern tropical Pacific, with the U.S. West Coast as one of the most important feeding areas during summer and fall. Estimates of blue whale abundance in the eastern North Pacific have been stable based on mark-recapture methods [23] and one hypothesis is that this stock has already reached carrying capacity [24]. Line-transect estimates of blue whale density and abundance along the US West Coast declined from the 1990s to 2000s [25,26], although this appears to be due to a shift in occurrence resulting from changing ocean conditions [27]. Along the U.S. West Coast, one humpback stock is currently recognized by the MMPA, with somewhat distinct feeding areas in California/Oregon and northern Washington/British Columbia [19,28]. Humpback abundance in both these areas has increased steadily through the 1990s and 2000s [19,23]. Humpbacks also face significant mortality from entanglement with fishing gear [15,29]. Fin whale population structure is less understood. Fin whales appear to be recovering [30], though current estimates are still well below 1974 estimates and even more significantly depleted from pre-whaling levels [31].

All three stocks were listed as Endangered under the Endangered Species Act with associated legal protections. In 2016 NOAA recognized 14 Distinct Population Segments (DPSs) of humpback whale under the Endangered Species Act based on a status review [32] with four DPS units recognized in the North Pacific, two of them staying endangered, one down listed to threatened, and one delisted. The West Coast stock of humpback whales is comprised of the Central American DPS, listed as endangered, and the Mexico DPS listed as threatened. The human-caused mortality limit (also known as Potential Biological Removal, PBR) for U.S. waters is set by the National Marine Fisheries Service at 2.3, 11 and 16 for blue, humpback and fin whales, respectively [33]. For humpback whales, current PBR is assessed at the stock level and not separately for each DPS.

Estimates of strike mortality to date have had to rely on 1) limited reports of ship strikes which are typically not observed or reported, and 2) records from stranded animals, which are known to dramatically underestimate the true number of deaths [34]. Blue, humpback and fin whales are often negatively buoyant, so that most carcasses sink upon death [35,36]. While some whale cadavers may remain floating or resurface from waters less than 100 meters deep as decomposition increases buoyancy, carcass detection is low due to sinking, scavenging and currents unfavorable to beaching [7,16]. Some analyses have compared spatial overlap between whales and vessels as an estimate of relative risk between management scenarios at local scales [16,21]. However, no comprehensive estimate of mortality exists and no analysis has been conducted covering all or most of population ranges in U.S. waters as our work does for three whale species.

Current efforts at mitigation focus only on the highly visible ship traffic lanes off San Francisco and Long Beach where risk of collisions is likely to be especially intense. However, no information exists regarding what proportion of total risk can be addressed in these areas nor where other regions of high risk occur. The current state of knowledge leaves several important gaps: 1) we still do not know with any precision how many whales are killed or where those deaths occur, 2) we lack a synoptic understanding of the problem and therefore cannot prioritize locations and approaches nor manage strike risk well on the scales where regulations are made and enforced, and 3) there is no way to set mortality limits for smaller jurisdictions such as National Marine Sanctuaries. To fill these important needs, we calculate strike mortality using a quantitative framework based on encounter theory [37,38] and allocate the results to local and regional jurisdictions.

Here we conduct a new assessment of mortality to blue, humpback and fin whales across the entire U.S. West Coast Exclusive Economic Zone (EEZ). We use Automated Identification System (AIS) data on ship locations and characteristics combined with species distribution models (SDMs) of whale density and multi-sensor electronic tag data on whale behavior to model the spatial distribution of ship strike mortality. In addition, we estimate total mortality for each of the three populations in U.S. West Coast waters. Compared to previous work, this large spatial approach gives a much-needed holistic perspective that allows assessment of the total impact of ship strikes on the study populations. In addition, our model adds spatial variation in four model parameters and we assess model sensitivity to a fifth (Table 1), improving the validity of comparisons across space. Our results enable the design of a suite of marine spatial plans and regulations that have the potential to mitigate mortality from vessel collisions on the west coast.

**Table 1:** Variables used in the strike risk model and their sources. We report the expected likelihood of spatial heterogeneity for each variable and whether that variability could be included in the model.

Model Variable	Source	Spatial heterogeneity	Spatial variability included
Vessel velocity	AIS data	yes	yes
Vessel draft	AIS data	yes	yes
Critical distance	AIS data	yes	yes
Whale velocity	electronic tags	yes	no
Time in strike zone	electronic tags	likely	partial
Probability of avoidance	McKenna *et al*. 2015	possible	no
Probability of mortality	Conn & Silber 2013	possible	no

## Methods

### Ship data

To calculate spatial patterns of ship speed, draft, count, and track distance, we used AIS data downloaded from MarineCadastre.gov, Bureau of Ocean Energy Management, and National Oceanic and Atmospheric Administration (2016) (http://marinecadastre.gov/ais/). AIS is an onboard navigation system which transmits information about a vessel’s movements and characteristics and is required by the International Maritime Organization (IMO) for all vessels over 300 gross tons and by the U.S. Coast guard for most vessels over 65 feet in U.S. waters. The data is collected by a network of stations and satellites and processed by the Coast Guard to remove duplicate records and faulty data. To best match both the seasonal timing of peak whale abundance and survey data collected on whale density and distribution, we used data for 6 months from June to December of 2014 excluding November. We used the most recent year available, 2014, so that results were as current as possible and because patterns of ship traffic have changed in recent years due to modification of fuel and vessel speed regulations [18,39]. Due to changes in AIS processing by U.S. Coast Guard in November 2014, the resulting data was incomplete and inaccurate, necessitating exclusion of this month. To account for removing the month of November, we instead included December data as the closest available proxy.

Data are available as ArcGIS file geodatabases which include information for each AIS transmission (e.g., vessel identifier, location and speed), data pertinent to a given voyage (e.g. draft) and static vessel characteristics (e.g. width). We first filtered the data to include only cargo, passenger and tug vessels that were underway and traveling faster than 1 knot. We also excluded vessels with draft of less than 1 meter to avoid potentially erroneous AIS data and remove vessels with low likelihood of lethally striking whales. We established a geographical grid covering the U.S. West Coast EEZ at a resolution of ~144 km^2^ (~12 km by ~12 km) per cell, resulting in 7,829 cells within the study area. We calculated ship speed, ship width and ship draft for each grid cell using ordinary kriging in ArcGIS 10.3 to interpolate AIS vessel reports across the study area. Parameter optimization was employed in the kriging models to minimize mean squared error of the predictions. We checked the resulting surfaces to ensure they remained within plausible limits for each parameter. No values were found to fall outside the ranges of the original AIS data, supporting valid kriging results. Kriging has the added advantage of producing estimates of prediction surface error which we used to provide error estimates for our model results.

We used the ArcGIS Track Builder tool (available from http://marinecadastre.gov/ais/) to create line representations of vessel tracks. We then clipped the tracks to our grid cells and used the resulting line features to calculate the number of unique voyages and the total track length for each grid cell. Track length was divided by mean vessel speed and multiplied by the number of vessels to give the total vessel transit time.

### Whale data

**Fig 1:**
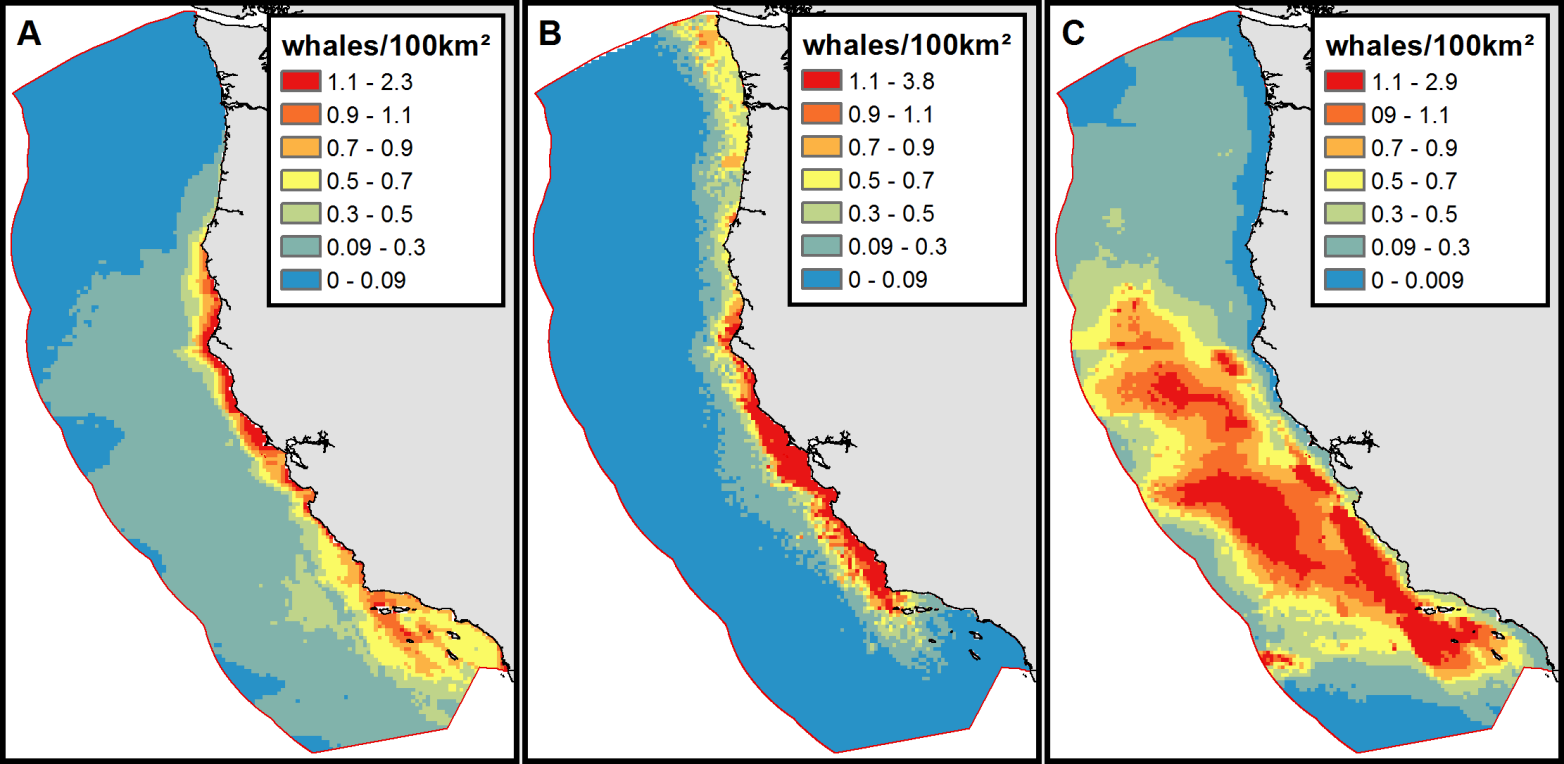
**Species Distribution Model density blue whales (A), humpback whales (B), and fin whales (C).** Density predictions derived from Becker et al. 2016. Note that to facilitate comparison across species, the scale breaks are the same with the exception of the top class maximum.

Whale density for blue, humpback and fin whales was sourced from SDM predictions. We adapted data from models built using environmental predictors and fit to whale sightings during line transect surveys of the California Current [40]. Surveys were conducted from July to December in 1991, 1993, 1996, 2001, 2005, and 2008 by the National Oceanographic and Atmospheric Administration and covered waters off California, Oregon, and Washington out to 300 Nm (555.6 km). Once models were selected, the authors predicted density from 8-day periods of environmental data to a grid of 10-km resolution cells. These predictions were averaged across the period of surveys to give a mean density representing the 1991–2008 average density during July to December. Full details of the model building, selection and prediction methods can be found in Becker *et al*. [40]. To match our data grid, we re-projected the rasterized the SDM data using bilinear interpolation in the *raster* package of the R statistical language [41]. We then converted whale density to whales per grid cell by multiplying the SDM density by the area of each grid cell (Fig 1).

We used data from 25 deployments of archival recording tags on all three species of whales off the U.S. West Coast in May to October of 2015 and 2016. We calculated the time-at-depth in 1 meter bins for blue, humpback and fin whales (S1 File). We used data from 13 tag deployments on blue whales, 9 on humpbacks and 3 on fin whales. Tags were either Wildlife Computers TDR-10 or Acoustimetrics Acousonde digital archival tags. We used almost 2,000 hours of data from deployments that were attached with short darts to maximize attachment durations [42]. This also provides a representative sample of mean behavior and to balances day-night differences in depth distribution [43]. Because of our specific interest in the proportion of time whales were spending in the upper portion of the water column, we corrected for tag pressure drift by recalibrating the tags to 0 depth every 30 minutes by setting the 10th highest reading to 0 for each period. This periodic zeroing of pressure data avoids errant spikes in the depth readings. We inspected the corrected dive data to insure this approach reasonably accounted for any drift. We then converted time-at-depth to cumulative proportion of time spent above each depth interval. Finally, we used the mean ship draft for each grid cell to calculate the proportion of time each species spent in the strike zone, equivalent to the probability of a whale being in the strike zone when an encounter occurs. Tests of strike dynamics between scale models of a right whale and vessel found that propeller strikes may occur with the model positioned at a depth up to two times the draft of the vessel [44]. However, while 100% of trials with whales at the surface resulted in strikes, 50% of trials at twice the vessel draft caused propeller strikes and the authors note that the rigid whale model may have accentuated this compared to the deformable nature of a real whale. We therefore chose to define the strike zone as the mean vessel draft to ensure conservative model results, but explored the effect of extending the strike zone to 1.5 and 2 times the mean draft following the results in Silber *et al*. [44] (S2 File).

### Model framework

To calculate realistic strike risk and to estimate mortality, we modify a quantitative framework based on encounter theory [37]. We incorporate the effects of ship characteristics and whale behavior to better quantify the resulting strike risk beyond measures of concurrent space use by ships and whales (e.g. [7,29]). We include spatial variation in three important vessel parameters—velocity, vessel draft and vessel beam—and use multi-sensor tag data to quantify whale time-at-depth, allowing us to calculate strike risk and mortality estimates across the U.S. West Coast EEZ. In addition, we explored the importance of whale velocity to model results using published ranges of swimming speed. The model can be summarized into three parts: encounter risk, strike risk and mortality estimation with each part predicated on the previous.

**Fig 2:**
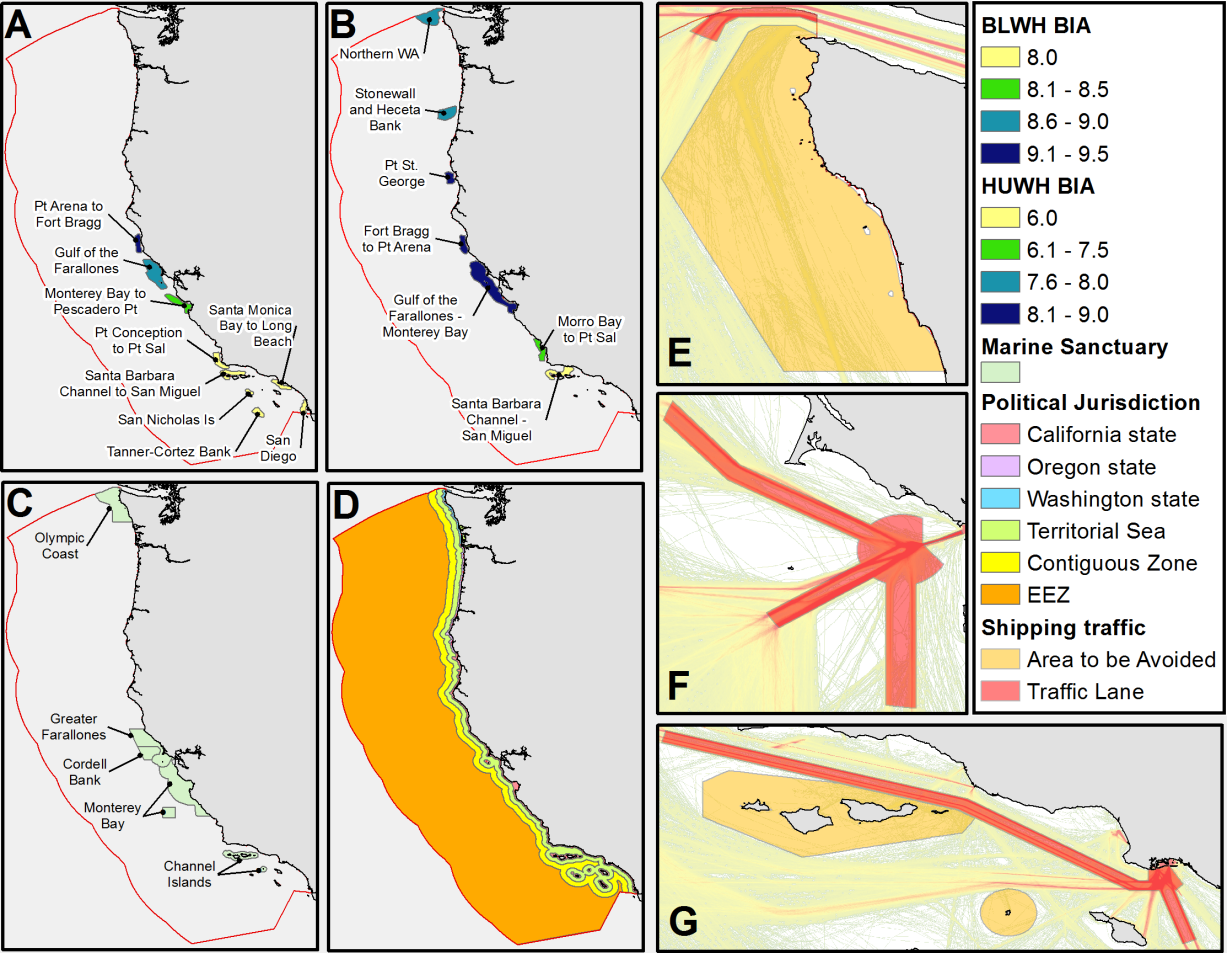
Jurisdictions used to assess the distribution of mortality. Biologically Important Areas (BIAs) for blue (A) and humpback (B) whales are from Calambokidis 2015. BIA color represents the mean of the months when whale presence occurs. Note that the scales are different for the two species. National Marine Sanctuaries (C) and political jurisdictions (D) including state waters (to 3 Nm; 5.6 km), territorial seas (to 12 Nm; 22.2 km), the Contiguous Zone (to 24 Nm; 44.5 km) and the Exclusive Economic Zone (to 200 nm; 555.6 km) are important for regulations on ship traffic and management of whales. Ship traffic schemes for northern Washington (E), the San Francisco Bay Area (F) and the Port of Long Beach and Santa Barbara Channel (G) are overlaid on 2013 ship density from Marine Cadastre.

The study area is waters under U.S. jurisdiction offshore from California, Oregon and Washington and covers an area of 811,936 square kilometers (Fig 2). The region includes important habitat and feeding areas for all three whale species [20] and covers most or all of the U.S. habitat of each stock in question. To apply the model we calculated model inputs for each grid cell of the study region. The risk of an encounter between whales and ships is a function of the velocities of whales and vessels, the surface area of the region in question, the distance traveled by vessels and the density of ships and whales. For any encounter that occurs, strike risk depends on the probability that whales are within the strike depth zone when an encounter occurs and the probability of collision avoidance. Finally, the mortality rate can be calculated from risk based on the probability of mortality from a strike. Mortality rate allows the estimation of deaths for a given area and time period depending on the density of whales and ships. Thus, this model framework improves significantly on previous risk estimates that rely solely on the densities of whales and ships as proxies for risk. We give an overview of the model here, but details on its derivation, structure and justification can be found in Martin *et al*. [37].

Briefly, a ship and whale can be represented as moving points within a defined space, in our case, each grid cell. If these points come within a threshold distance (termed the critical encounter radius, *r*_*c*_), an encounter is considered to occur. For our model, the critical radius is defined as

$$r_{c}=Vessel\;Width+\sqrt{\frac{Total\;Length\times Head\;Width}{\pi }},$$

representing the most common case that the ship will approach the whale bow first given the much faster speeds of the ship relative to the whale. Total length and head width for each whale species was taken from the literature and where multiple data sources were available, measurements were averaged. Values used were 2.96 m width [45] and 20.9 m length [46] for blue whales, 3.21 m width [47] and 13.5 m length [48] for humpback, and 2.65 m width and 18.48 m length for fin whales [49].

The encounter rate, *λ*_*e*_, between one whale and one vessel in each area was calculated as

$$\lambda_{e}=\frac{2r_{c}}{S}\int_{v_{m}}I(v_{m},v_{b})v_{m}dv_{m},$$

where *r*_*c*_ is the critical radius, *S* is the area, *v*_*m*_ is the whale velocity, *v*_*b*_ is the vessel velocity, and *I*(*v*_*m*_, *v*_*b*_) is an increasing function of the velocities as derived from encounter theory. While Martin et al. [37] used an estimated probability function for animal velocity, we instead used mean velocities that were calculated from satellite tags and reported in the literature for each species [50–54]. Because these values were based on satellite data from a limited number of individuals and swimming speeds can vary depending on behavioral state [50,54], we tested model sensitivity to a range of realistic swim speeds. In addition, we wanted to determine the possible impact that omitting spatial variation in swim speeds could have on mortality estimates. We therefore produced additional model results using swimming velocities reported for whales during directed travel (e.g., migration) and during ‘area restricted searching’, often associated with feeding behavior (Text and Tables B and C in S2 File).

Encounters do not necessarily represent a collision, but rather the co-occurrence of a vessel and whale in both space and time. Encounters only result in collision if 1) the whale is within the top part of the water column where the vessel hull penetrates (the strike zone) and 2) the whale and vessel fail in any collision avoidance behavior. The product of the encounter rate, the probability the whale is in the strike zone and the probability of *not* avoiding collision gives the expected number of collisions. Because the majority of vessels represented in our model are large tanker and cargo vessels with limited visibility and poor reaction capability, we consider avoidance behavior by vessels to be close to zero as was found for large cruise vessels [55]. We parameterized collision avoidance by whales under three scenarios: decreasing avoidance with increasing vessel speed (Model 1), constant 55% avoidance (Model 2), and no avoidance (Model 3) (Text and Figure A in S2 File).

Avoidance behavior of whales to ships is poorly understood and likely varies by species. Evidence from analyses of whale ship strikes on the east coast has shown that strike mortality increases with increasing ship speed [10]. While various mechanisms for this relationship have been proposed (e.g. [44]), the decreased lethality of ship strikes at slower speeds has been best documented[13]. However, some research has also found that faster vessels lead to closer encounters and decreases in whale avoidance [4,10,55]. Both linear [10] and non-linear [55] negative logistic relationships have been found between avoidance and ship speed. We therefore formulated a model with a low-slope logistic function with inflection at 11.8 knots, the change-point in encounter distance identified by Gende [55].

McKenna *et al*. [12] reported in a study of encounters between whales and vessels that in 55% of observations, an avoidance dive was initiated. This measure does not represent whale responses to impending collision and so is used as a hypothetical more conservative second model of avoidance. Though unlikely in reality, this model formulation assumes that all animals that initiate such dives, successfully avoid the vessel. In contrast, the same study found that blue whales may surface in response to approaching vessels [12], putting them at greater risk of collision. In addition, McKenna *et al*. [12] found that for whales at the surface to avoid collisions, they must initiate response dives when vessels are a quarter to a half a kilometer away. Furthermore, right whales showed no behavioral response to approaching vessels or playback of equivalent sounds [56]. Given that some whale response behavior may place individuals at greater (not less) risk and that observed avoidance responses by both ships and whales appear to be of limited efficacy [12,55], we also include a more extreme no avoidance scenario. So, we formulated three alternate avoidance parameterizations based on behavioral observations and the first quantification of responses measured by tags.

Not all collisions will result in the death of the whale. To calculate mortality, we must include the probability of mortality given a collision. Conn and Silber [10] used empirical data on ship strikes to fit a mathematical relationship between ship speed and probability of mortality. We use this equation and vessel speed from AIS to calculate the mean probability of mortality for each grid cell. Using these model components we calculated mortality for each grid cell as

$$Mortality=\lambda_{e}tP(Strike\;depth)(1-P(Avoidance))P(Mortality|v_{b})N_{m}N_{b},$$

where *t* is the total time of vessel transits, *P*(*Strike depth*) is the probability the whale is within the mean vessel strike depth, (1−*P*(*Avoidance*)) is the probability of no successful avoidance, *P*(*Mortality*|*v*_*b*_) is the probability of mortality given mean vessel speed, and *N*_*m*_ and *N*_*b*_ are the number of whales and boats, respectively.

This model was applied to each grid cell for each month and species combination. Monthly results were summed to create spatially-explicit predictions of mortality. Because many of the model parameters are either temporal averages or characteristic mean values, results for individual months are unlikely to be representative of true monthly mortality. Instead, the model predictions reported represent sums for the 6-month peak whale period (July-December) within the study area. We assume that these values represent the majority of strike mortality for the whole year, especially for blue whales, since all three species show decreased extent and abundance during Winter and Spring compared to Summer and Fall [57–59]. While fin and humpback whales are present in the U.S. EEZ outside of July-December, abundances are lower and whales tend to be found further offshore where ship density is lower [58–60].

### Stranding records

We obtained west coast stranding records attributed to ship strike mortality from the NOAA National Marine Mammal Stranding Network for 2006–2016. For records where latitude and longitude were not reported, we used geocoding of the record location or description information to assign geographic coordinates. We subsequently binned stranding records for each species into 15 equal latitudinal bins of approximately one-degree each and calculated the total number of mortalities per bin. These stranding data were plotted along with maps of mortality to provide an empirical comparison for spatial patterns.

If a struck whale carcass is transported a significant distance on the bow of a ship, even coarse-scale binning such as we use may be skewed by the associated stranding records. We therefore use descriptions of strandings to determine if 1) a record identifies that the carcass was found or seen on the bow of a vessel and 2) if the stranding was detected in or near a port where it is unlikely a carcass would be found due to natural transport by winds and currents. By identifying these records, we provide an estimate of where transport by vessels may be influencing stranding prevalence.

### Spatial statistics and translation to management targets

For each of the avoidance models, we calculated total mortality across the study area to compare results and assess model sensitivity. For Models 2 and 3, avoidance was parameterized as a static scalar while Model 1 avoidance varied across space with vessel speed. Therefore, relative spatial patterns of mortality were identical for Models 2 and 3. In addition, visual examination of the difference between Model 1 and Model 2 mortality maps showed insignificant disparity in spatial patterns. Therefore, for all subsequent calculations of relative mortality across space, we report only values for the most conservative model, Model 2.

To inform strategies for regulating and mitigating ship strikes, we determined the areas where mortality was above 1) the mean and 2) the 90^th^ percentile. We converted these areas to polygons and determined the total area covered by each, the sum of mortality within the area and the proportion of total mortality represented.

A number of management jurisdictions are relevant to current and potential regulation of vessels to decrease ship strike mortality. We calculated statistics for these management jurisdictions as well as ecologically relevant areas (Fig 2). Regions included Biologically Important Areas (BIAs) for blue and humpback whales (no BIAs are defined for fin whales), vessel TSSs, National Marine Sanctuaries (NMSs), state and federal jurisdictions and areas on and off the continental shelf, an important oceanographic feature for whales. For the latter regions, we defined the continental shelf as the area <200 m depth and off-shelf as >200 m. BIA polygons were retrieved from the NOAA Ocean Noise Strategy website (http://cetsound.noaa.gov/important), TSSs and federal jurisdictions were produced by the NOAA Office of Coast Survey (downloaded from https://data.noaa.gov/dataset/shipping-fairways-lanes-and-zones-for-us-waters and http://www.nauticalcharts.noaa.gov/csdl/mbound.htm, respectively), NMS boundaries were from the National Marine Sanctuaries GIS data page (http://sanctuaries.noaa.gov/library/imast_gis.html), and state jurisdictions were obtained from Marine Cadastre (http://marinecadastre.gov/data/). For each jurisdiction, we calculated the total mortality, mortality per 100,000 km^2^ and proportion of total mortality. Mortality per area gives a measure of the intensity of risk while proportion of total mortality provides a gauge of the relative importance of vessel strikes within the given area.

## Results

We report summary values and spatial patterns of our model results here and compare them to documented stranding records. For simplicity, we refer to the model results as ‘mortality’ and ‘strike intensity’ (deaths per 10^4^ km^2^), but it is important to note that except for discussion of stranding records, these values represent modeled estimates of ship strikes, not observed whale mortalities.

### Study area mortality and strandings

**Fig 3:**
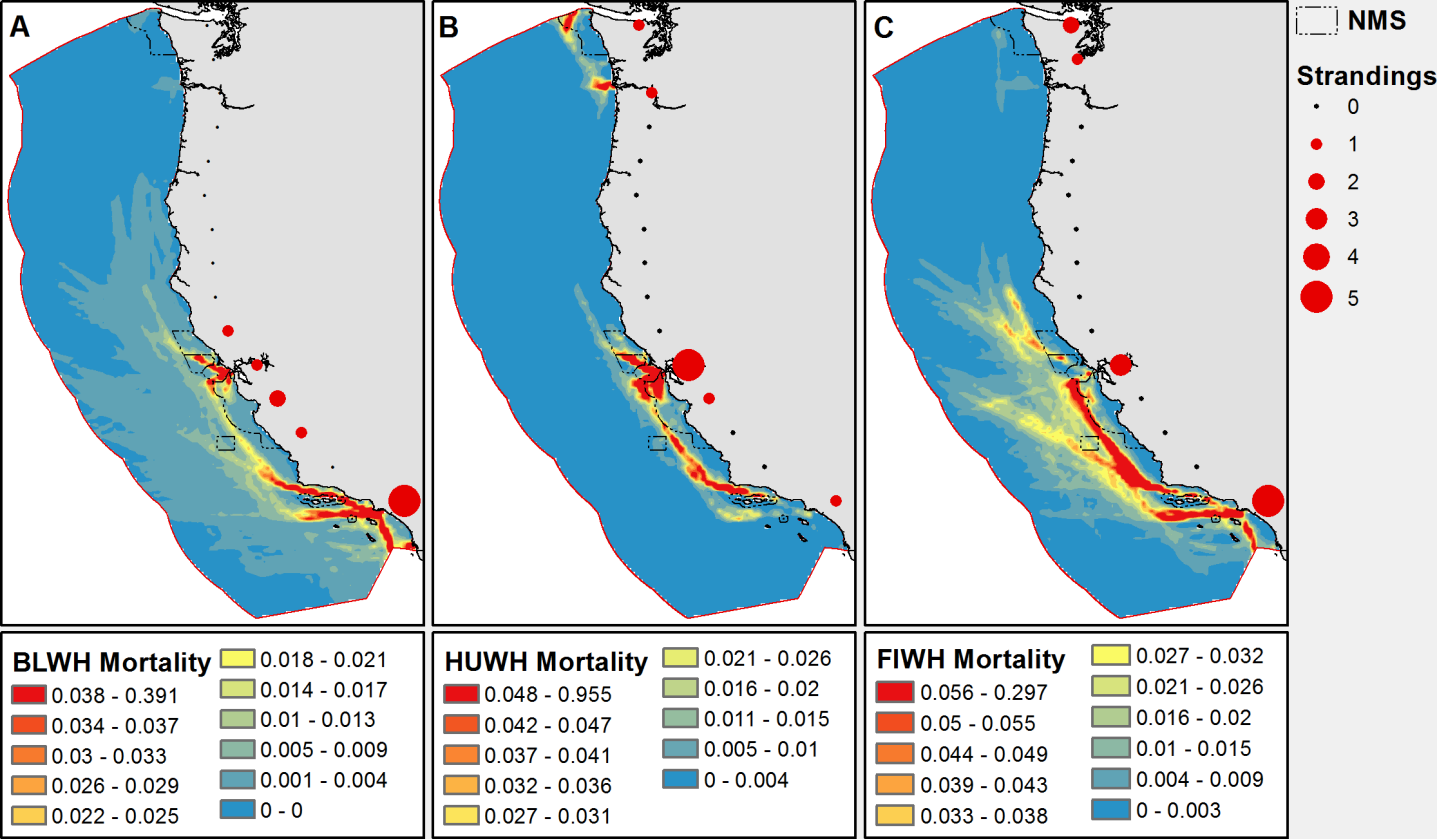
**Smoothed distribution of blue (A), humpback (B), and fin whale (C) mortality estimated using Model 2.** Warmer colors represent higher mortality and values are predicted mortalities per ~144 km^2^ grid cell for the 6-month study period. Red circles represent latitudinally binned ship strike stranding records from 2006–2016. Dashed lines are National Marine Sanctuary boundaries.

Total fin whale mortality across the study area is approximately twice that for blue and 2.4 times humpback whale mortality (Table 2). Mortality results for the limit scenario of no avoidance (Model 3) are approximately twice estimates using a constant avoidance rate of 55% (Model 2) and speed-dependent avoidance (Model 2). Regardless of model, mortality estimates are significantly greater than PBR limits for all three species. Compared with PBR, Model 2 estimates are 7.8, 2.0, and 2.7 times as great. For all three species, mortality is greatest along the coast of central and southern California with swathes of high mortality occurring along shipping routes between the port of Long Beach/L.A. and the San Francisco Bay Area (Fig 3).

**Table 2:** Total study area mortality estimates (July-December) for each species and three different models incorporating different estimates of collision avoidance. Potential Biological Removal (PBR) limits (annual) are included for each species for reference. Stranding extrapolations (annual) are based on 5% (best) and 17% (low) carcass recovery rates.

Species	PBR	2006–2016 Strandings	Stranding Extrapolation best(low)	Model	Total Mortality
Blue whale	2.3	10	20(5.9)	1) Decreasing with speed	18
				2) 55% avoidance	18
				3) No avoidance	40
Humpback whale	11	14	28(8.2)	1) Decreasing with speed	22
				2) 55% avoidance	22
				3) No avoidance	48
Fin whale	16	11	22(6.4)	1) Decreasing with speed	46
				2) 55% avoidance	43
				3) No avoidance	95

PBR limits are annual, while our model results are for the peak whale period from July-December. This difference may result in some underestimation in our modeled mortality relative to PBR guidelines. However, for humpback and blue whales, PBR accounts for the approximate proportion of time these species spend outside the EEZ (1/2 and 3/4, respectively) [33], supporting a reasonable comparison with our 6-month study period. Conversely, because fin whale movements and distributions are not well-understood, PBR includes no proportional adjustment, though there is evidence of lower abundance in winter and fall [58,59]. Thus, while comparisons between our model results and PBR should be made cautiously, they are likely justified for blue and humpback whales and are conservative (PBR may be elevated given no temporal adjustment) for fin whales.

Additional higher mortality regions for blue and fin whales (especially the latter) extend offshore along major trans-Pacific shipping routes. In contrast, humpback whales are the only species with significant areas of high mortality off of northern Oregon and Washington. Maximum mortality per grid cell (~144 km^2^) is 0.39 for blue whales, 0.96 for humpbacks and 0.30 for fin whales. The higher maximum for humpback whales highlights the greater intensity of mortality across a smaller area as compared to the other two species. Fin whales show the most widespread and furthest off-shore mortalities, while humpback mortality levels are coastally compressed, matching their distribution patterns (Fig 3).

Records of stranding mortalities were highest in bins with large ports, though it is important to note the limitations of stranding records which may not represent the actual location of death. The National Marine Mammal Stranding Network data comprised 35 total records; 10 blue whale, 11 humpback whale and 14 fin whale ship strike strandings. Five mortalities occurred in the Long Beach/L.A. bin during the data period for both blue and fin whales. In the San Francisco bin, stranding mortality was 5 and 3 whales for humpback and fin whales, respectively. Contrary to the other species, fin whales also had 3 mortalities in the furthest north bin, which include records from Seattle and the Puget Sound region. No vessel strike strandings for any of the species were detected along the stretch of coastline between Point Arena, California and Portland, Oregon.

Out of a total of 35 ship strike stranding records, 5 (14%) contained mention that the carcass was transported by a ship and 3 (8.6%) were detected in the waters of an enclosed port suggesting they were also moved by vessels. Fin whales accounted for all of the carcasses that were transported on ships and 2 out of 3 found in ports. Thirty-six percent (5 of 14) of fin whale strandings were detected on vessel bows and 14% (2 of 14) were found in ports. The other port record, a blue whale carcass, was found in Long Beach Harbor. One fin whale was brought in to the Port of Tacoma, Washington on the bow of a ship, while two were brought to Port of Oakland and two to ports in Los Angeles County. All three carcasses in an enclosed port were in the Los Angeles area; fin whales were found in Hueneme and Los Angeles Harbors and the blue whale was found in Long Beach Harbor.

**Fig 4:**
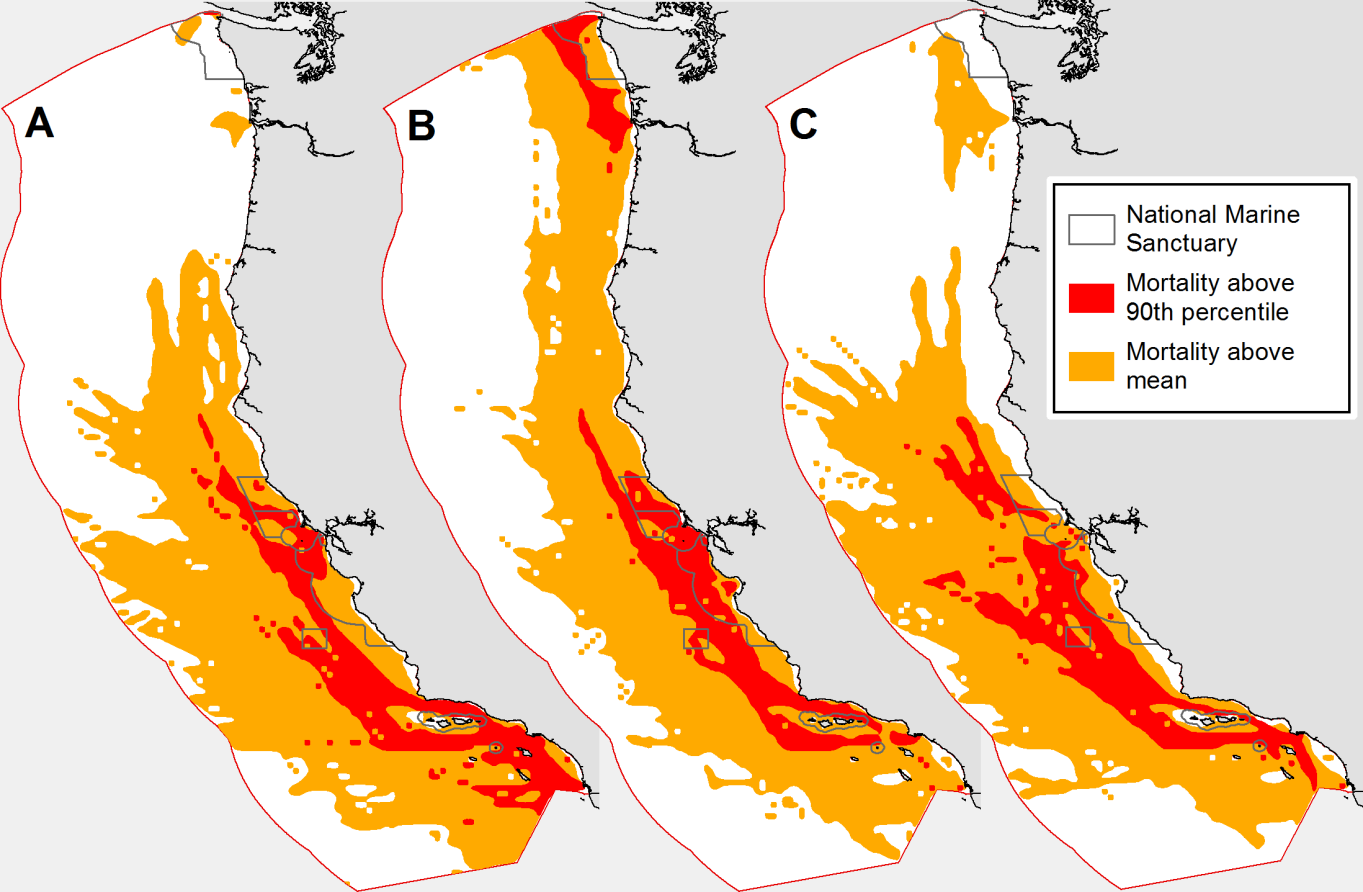
Areas classified as above the study area mean (orange) and greater than the study area 90^th^ percentile (red). Panels depict whale mortality for blue (A), humpback (B), and fin (C). Dashed lines represent the National Marine Sanctuaries.

The map of mortality above the mean highlights the difference in distribution between more coastal humpbacks and wider-ranging blue and fin whales (Fig 4). For blue and fin whales, mortality levels above the 90^th^ percentile were confined to waters off California while humpback whales have a large high mortality area off Oregon and Washington. Unsurprisingly, mortality above the 90^th^ percentile is concentrated along major shipping routes and in TSSs, but the majority of these areas remain well offshore except by the major California Ports and in the Santa Barbara Channel. Areas above the mean include more than 98% of mortality for all three species (Table 3). The area above the 90th percentile (covering ~10% of the study region) contained 74%, 82% and 65% of mortality for blue, humpback and fin whales, respectively. Thus, for all three species the vast majority of strike mortality is found in 10% of the total study area. This is most true for humpbacks where nearly 82% of mortalities are found in the region above the 90^th^ percentile.

**Table 3:** Comparison of total mortality and mortality within the 50^th^ percentile and 90^th^ percentile areas.

		50th percentile area		90th percentile area	
species	Total mortality	Mortality	Percent of total mortality	Mortality	Percent of total mortality
Blue whale	18	18	98%	13	74%
Humpback whale	22	20	93%	18	82%
Fin whale	43	42	98%	28	65%

### Ecologically significant regions and political jurisdictions

Biologically Important Areas (BIAs) identify regions of ecological importance and elevated whale abundance [20]. For blue whales, the highest intensity of mortality is in the Santa Monica Bay to Long Beach BIA, with 51 mortalities per 10^4^ km^2^ accounting for over 3% of total mortality (Table 4). Other BIAs with elevated strike intensity include Santa Barbara Channel to San Miguel (22 mortalities/10^4^ km^2^), San Diego (20 mortalities/10^4^ km^2^) and Gulf of the Farallones (14 mortalities/10^4^ km^2^). In contrast, the Gulf of the Farallones BIA accounts for the greatest portion of mortality at 4%, followed by Santa Monica Bay to Long Beach (3%) and Santa Barbara Channel to San Miguel (2%). San Nicholas Island and Point Arena to Fort Bragg BIAs are relatively free of strike risk, each accounting for much less than 1% of total mortality. In combination, the blue whale BIAs encompass just 2% of the study area but 13% of total mortality. Because the data underlying the BIAs is finer-scale and coastally focused, while the surveys used to predict density are further offshore and coarser resolution, our mortality predictions for these small areas may have elevated error.

**Table 4:** Statistics of blue whale strike mortality within the Biologically Important Areas (BIAs) defined in Calambokidis et al. 2015. Total mortality, mortality per 100,000 km^2^ and percent of total study area mortality are reported for each BIA.

Name	BIA Area (km^2^)	Months of peak abundance	Area Mortality	Mortality per 10^4^ km^2^	Percent of total mortality
Gulf of the Farallones	5,243	July—November	<1	14	4%
Monterey Bay to Pescadero Pt	2,378	July—October	<1	4	<1%
Santa Barbara Channel to San Miguel	1,981	June—October	<1	22	2%
Pt Conception to Pt Sal	1,743	June—October	<1	9	<1%
Pt Arena to Fort Bragg	1,419	August—November	<1	1	<1%
Santa Monica Bay to Long Beach	1,187	June—October	<1	51	3%
Tanner-Cortez Bank	1,076	June—October	<1	5	<1%
San Diego	984	June—October	<1	20	1%
San Nicholas Is	427	June—October	<1	3	<1%

In contrast, the mortality in humpback whale BIAs is greatly skewed to the Gulf of the Farallones-Monterey Bay BIA, which accounts for 17% of total humpback strike mortality (Table 5). The second most impacted BIA is the Santa Barbara Channel-San Miguel area with 2% of total mortality but a relatively high strike intensity of 15 mortalities/10^4^ km^2^. The percent mortality within all humpback whale BIAs is 21% while their area is 3% of the total study area.

**Table 5:** Statistics of humpback whale strike mortality within the Biologically Important Areas (BIAs) defined in Calambokidis et al. 2015. Total mortality, mortality per 100,000 km^2^ and percent of total study area mortality are reported for each BIA.

Name	BIA Area (km^2^)	Months of peak abundance	Area Mortality	Mortality per 10^4^ km^2^	Percent of total mortality
Gulf of the Farallones—Monterey Bay	9,761	July—November	3	32	17%
Northern WA	3,393	May—November	<1	6	1%
Stonewall and Heceta Bank	2,573	May—November	<1	<1	<1%
Santa Barbara Channel—San Miguel	2,639	March—September	<1	15	2%
Morro Bay to Pt Sal	1,908	April—November	<1	2	<1%
Fort Bragg to Pt. Arena	1,591	July—November	<1	2	<1%
Pt St. George	1,233	July—November	<1	<1	<1%

Current efforts to address ship strike mortality have focused on the shipping lanes off San Francisco and Long Beach. Risk levels are significantly higher in these vessel TSSs due to the high intensity of ship traffic (Table 6). Strike intensity is the highest in the San Francisco TSS for humpbacks out of any of the ecological or jurisdiction areas considered (255 deaths/10^4^ km^2^). Blue whale strike intensity is similar for San Francisco (65 deaths/10^4^ km^2^) and Southern California (61 deaths/10^4^ km^2^) TSSs, but the latter accounts for 8% of mortality compared to 4% for the former. Fin whale strike intensity is 48 deaths/10^4^ km^2^ in the Southern California TSS, but only accounts for 3% of mortality. While strike intensity is high for all three species in both the San Francisco and Southern California TSSs, the combined mortality for these two areas represents just 12% (blue whale), 17% (humpback whale) and 3% (fin whale) of total mortality. While strike intensity is high, the percent of fin whale mortality is minor in both California lanes. For both blue and humpback whales, strike intensity is moderate for the Washington lanes, but due to the small area within the study area, percent mortality is negligible.

**Table 6:** Mortality statistics for shipping lanes. Mortality per 100,000 km^2^ and percent of total study area mortality are also reported for each region.

		Blue Whales			Humpback Whales			Fin Whales		
Region	Area (km^2^)	*Area Mortality*	*Mortality per 10*^*4*^ *km*^*2*^	*Percent of total mortality*	*Area Mortality*	*Mortality per 10*^*4*^ *km*^*2*^	*Percent of total mortality*	*Area Mortality*	*Mortality per 10*^*4*^ *km*^*2*^	*Percent of total mortality*
San Francisco	1,136	1	65	4%	3	255	13%	<1	20	<1%
Southern California	2,314	1	61	8%	<1	36	4%	1	48	3%
Washington	166	<1	4	<1%	<1	6	<1%	<1	<1	<1%
Waters outside lanes	798,015	16	2	86%	18	2	81%	41	5	96%

Within all NMSs except the Olympic Coast, strike intensity is moderate to high while percent of mortality varies significantly among sanctuaries (Table 7). NMSs are especially important regions of risk for humpbacks, with strike intensity as high as 49 deaths/10^4^ km^2^ and percent total mortality reaching 19%, both within the Greater Farallones NMS. Metrics are moderate in all sanctuaries for blue whales, with strike intensity ranging from less than 1 deaths/10^4^ km^2^ in the Olympic Coast NMS to 14 deaths/10^4^ km^2^ in the Greater Farallones and the combined percent mortality for all NMSs equaling 16%. At opposite ends of the spectrum, fin whale combined NMS percent mortality is 7% while humpback percent mortality sums to 42%. The majority of humpback mortality was within the Greater Farallones (19%) and Monterey Bay (13%) NMSs. Of the three species, only humpbacks face significant strike intensity (10 deaths/10^4^ km^2^) or percent mortality (4%) in the Olympic Coast NMS.

**Table 7:** Mortality within National Marine Sanctuaries. Mortality per 100,000 km^2^ and percent of total study area mortality are also reported for each sanctuary.

		Blue Whales			Humpback Whales			Fin Whales		
National Marine Sanctuary	Area (km^2^)	*Area Mortality*	*Mortality per 10*^*4*^ *km*^*2*^	*Percent of total mortality*	*Area Mortality*	*Mortality per 10*^*4*^ *km*^*2*^	*Percent of total mortality*	*Area Mortality*	*Mortality per 10*^*4*^ *km*^*2*^	*Percent of total mortality*
Cordell Bank	3,331	<1	12	2%	1	34	5%	<1	10	<1%
Channel Islands	3,818	<1	13	3%	<1	6	1%	<1	10	<1%
Greater Farallones	8,548	1	14	7%	4	49	19%	<1	7	1%
Monterey Bay	15,795	<1	5	4%	3	18	13%	2	10	4%
Olympic Coast	8,259	<1	<1	<1%	<1	9	4%	<1	<1	<1%

As evidenced by the distribution of risk in Fig 3, the majority of strike mortality is predicted off the coast of California, a pattern mirrored in the risk metrics of the state submerged lands jurisdictions (Table 8). Mortality in Oregon and Washington waters is zero or negligible for all three species, though it is slightly higher for humpbacks. In California, both blue (5 deaths/10^4^ km^2^) and humpback (4 deaths/10^4^ km2) risk intensity is moderate and percent mortality is 4 and 3 percent, respectively. The more offshore distribution of fin whales keeps intensity and percent mortality low, even in California waters. This same pattern is revealed for fin whale strikes on the continental shelf (2 deaths/10^4^ km^2^) vs. off (6 deaths/10^4^ km^2^) (Table 9). While the majority of mortality occurs off the shelf for all three species simply due to the vastly larger area off the shelf, the percentages are much closer for humpbacks at 36% on the shelf and 64% off the shelf. Given the disparity in areas, the nonetheless significant mortality on the shelf for both blue and humpback whales highlights their tendency to preferentially utilize waters near or on the continental shelf.

**Table 8:** Mortality within sovereign waters (3 Nm offshore) for each of the three west coast states, California, Oregon and Washington. Mortality per 100,000 km^2^ and percent of total study area mortality are also reported for each state.

		Blue Whale			Humpback Whale			Fin Whale		
Jurisdiction Name	Area (km^2^)	*Area mortality*	*Mortality per 10*^*4*^ *km*^*2*^	*Percent of total mortality*	*Area mortality*	*Mortality per 10*^*4*^ *km*^*2*^	*Percent of total mortality*	*Area mortality*	*Mortality per 10*^*4*^ *km*^*2*^	*Percent of total mortality*
California	14,280	<1	5	4%	<1	4	3%	<1	2	<1%
Oregon	3,268	<1	<1	<1%	<1	2	<1%	<1	<1	<1%
Washington	2,049	<1	<1	<1%	<1	2	<1%	<1	<1	<1%

**Table 9:** Mortality on and off the continental shelf (defined by the 200-meter isobath). Mortality per 100,000 km^2^ and percent of total study area mortality are also reported for each region.

		BLWH Mortality			HUWH Mortality			FIWH Mortality		
Area Name	Area (km^2^)	*Area mortality*	*Mortality per 10*^*4*^ *km*^*2*^	*Percent of total mortality*	*Area mortality*	*Mortality per 10*^*4*^ *km*^*2*^	*Percent of total mortality*	*Area mortality*	*Mortality per 10*^*4*^ *km*^*2*^	*Percent of total mortality*
Continental shelf	57,030	3	5	15%	8	14	36%	1	2	3%
>200m	754,905	15	2	85%	14	2	64%	41	6	97%

### Model sensitivities

Because of uncertainty in model parameters and omission of potential sources of spatial variability, we tested the sensitivity of model results to a range of whale swimming speeds and two alternate definitions of the strike zone. Details of each test can be found in Supplement 1, but we give a brief account of results here. Tests of swim speeds corresponding to mean, traveling and area restricted search velocities were conducted for all three species. Total mortality estimates were remarkably insensitive to these plausible ranges of swim speed, with results differing by less than 1.2 deaths and 1.3% in all permutations (Text and Table A in S2 File). In addition to confirming that any inaccuracy in swim speed parameterization will have negligible effect on strike mortality, these results suggest that including spatial variation in swim speed is not important for accurate ship strike results.

Changing the strike zone from one vessel draft to 1.5 or 2 times the vessel draft resulted in differences in mortality estimates up to 8.6 deaths greater for 1.5x and 15.9 deaths greater for twice the draft (Table A in S2 File). These increases in mortality estimates correspond to 20% and 37% larger estimates. Using twice the vessel draft increased mortality estimates by a minimum of 17% while 1.5x the draft increased death estimates by at least 10%.

## Discussion

Ship strike mortality is thought to be the number one killer of blue and fin whales and the second greatest cause of death for humpback whales along the U.S. West Coast [15]. Our results add to the growing evidence that ship strikes are an important source of mortality to whales in this region [6,12,21] and result in a death toll significantly above the Potential Biological Removal (PBR) limits set under the MMPA [16]. Spatial patterns of mortality from our model (Fig 3) align well with stranding records from 2006–2016 but reveal additional patterns of vital importance to effective ship strike mitigation. In particular, while strike patterns indicate that the highest mortality risk is in TSSs near large ports, a much larger proportion of risk occurs in waters outside these areas. By modeling ship strikes across the U.S. West Coast EEZ, we cover most of the range of blue, humpback and fin whale stocks under U.S. responsibility and provide a synoptic view of an important marine mammal conservation challenge.

The only other current source of total mortality estimates for west coast whales are extrapolations using strike records and carcass detection rates [16](Table 1). Fine scale spatial patterns of stranding records are not necessarily reliable indicators of strike locations. This is because carcasses can be carried away from the strike location by currents and winds before detection or be carried on the bow of a ship in which case they are often noticed only when the vessel reaches port. Our results suggest transport on vessel bows may have a large effect for fin whales, but identified few cases where this occurred for blue or humpback whales. Because of whales sinking, ocean currents and carcass decomposition, the rate of recovery for struck whales is very low. Recovery rates specific to blue, humpback and fin whales are unknown, so proxies from other species must be used.

Carcass recovery rates have been estimated for various cetacean species including 17% [61] for right whales, 6.5% for killer whales [62], <5% for grey whales [63], and 3.4% for sperm whales [34]. Right whales are the most buoyant whale species and thus provide a conservative limit scenario for extrapolation [64,65]. Sperm whales have been shown to have negative tissue buoyancy, but positive total buoyancy near the surface [66] so that recovery rates of sperm whales depend on lung inflation upon mortality with whaling records indicating that most float at death [64]. In contrast, blue whales appear to be negatively buoyant at or near the surface given that gliding decent begins at relatively shallow depths [67]. Thus, sperm whales (which have the lowest recovery rate) as well as grey whales are more likely than blue whales to float when deceased [64,65,68]. To produce an improved recovery estimate relative to the right whale rate, we use the average of the sperm, grey, and killer whales. Given that the available evidence [36,64–66,68] suggests the buoyancy of the study species is similar (humpbacks) or less (blue and fin) than these species, this ‘best’ estimate provides a better proxy. Using a high recovery rate of 17% to produce minimum strike estimates and 5% recovery (the mean of grey, killer and sperm whales) as a best estimate, we extrapolated ship strike mortality from 2006–2016 stranding data (Table 2). The blue whale stranding rate of 1.0 whales/yr. extrapolates to minimum 5.9 mortalities with a best estimate of 20. There were an average of 1.4 humpback strike strandings per year during the last decade, which extrapolates to a minimum 8.2 and best estimate 28 deaths. Fin whales had 1.1 strike strandings per year, leading to minimum 6.4 and best estimate of 22 deaths by extrapolation.

Comparing the extrapolated values to estimates from our models, we find close agreement with our most conservative model (Model 2) results (Table 2) for blue and humpback whales. Fin whale mortality estimates, however, were nearly twice the extrapolated value from strandings and significantly higher than blue or humpback mortality. These comparatively higher rates for fin whales result from both the larger population as well as the offshore distribution that overlaps significantly with several major shipping routes for a much greater spatial extent (Fig 1). Moreover, since fin whales are often found further offshore, beaching of dead carcasses from ship strikes may be less common, which would also explain the higher proportion of stranding records associated with transport on vessel bows compared to the other species. Alternately, there is some evidence that fin whale behavior allows for greater vessel avoidance [69] compared to blue whales and than the rates used in our model, which would have resulted in an inflated model prediction.

Model 1, which has the best theoretical support, produces estimates 8% and 22% smaller, and 94% greater than extrapolations for blue, humpback and fin whales, respectively. Model 3 (upper limit, no avoidance model) estimates are two times extrapolated mortality for blue whales, 73% greater for humpbacks and over 4 times as large for fin whales. These estimates provide important context for results from Monnahan *et al*. [24] which suggest ship strikes are not a limiting factor for blue whale population growth. The authors proposed that 10 blue whale strikes per year was a likely level in 2013, yet our most conservative estimate is nearly twice as great. Neither blue whale mortality levels from Model 1 nor 2 negate the results of the Monnahan *et al*. [24] model, but our higher predicted strike levels suggest caution is in order for results based on 10 strike deaths per year. If strike levels are indeed greater than used in that analysis, as our models suggest, the conclusion that collision mortality is not impacting the blue whale stock will be vulnerable to incorrect estimates of past population trends, carrying capacity or future trends in ship traffic. Additionally, their model is based on an assumption that there have been no changes in environmental conditions or threats from other factors during the near century-long period that their population model covers. Changes in blue whale distribution in the eastern North Pacific have been noted in apparent response to ocean regime shifts that occurred between the 1990s and 2000s [27]. Other anthropogenic activities such as Navy mid-frequency sonar only emerged in the latter half of the 20^th^ century and have been shown to alter blue whale feeding behavior [35,70].

During our model construction, we explicitly chose parameters and functional forms to provide a conservative estimate of total mortality. In addition, where significant uncertainty remains regarding the model formulation or appropriate parameter ranges, we explored model sensitivity to a range of plausible inputs (strike zone definition, whale swimming speed and functional form of avoidance). We caution that the use of numerical results for management purposes should include consideration of possible directional bias associated with these uncertainties. Because of our restrained model formulation, we believe that Model 2 results represent a low estimate of total strike mortality with associated implications for legal protections and necessary regulatory changes. Moreover, our models represent a long-term average and do not account for seasonal and inter-annual variation in whale density and distribution or trends in ship numbers or characteristics. When improved data become available, a temporally-explicit evaluation of strike risk would have significant management benefits. Alternately, the spatial parameters of the model are derived from empirical data, making conclusions regarding relative mortality across space robust. These spatial comparisons provide the most management value by identifying priority areas and shortcomings of current management approaches.

Current efforts to mitigate ship strikes on the west coast are limited to the TSSs in the Southern California Bight and outside the San Francisco Bay Area. Indeed, these areas show the highest intensity of mortality for all three whale species (Table 6). While the relatively small area covered by the TSSs makes them most tractable for regulation, it also means that the proportion of mortality represented is small. Under the hypothetical scenario where 90% of strike mortality was eliminated in all three TSSs, remaining Model 2 mortality would total 16, 18, and 41 deaths for blue, humpback and fin whales, respectively. Therefore, while regulation in shipping lanes is an important component of ship strike mitigation and a logical starting place, if limited to the TSSs, even the most successful regulation will not be sufficient to decrease ship strikes to anywhere near PBR levels.

Mortality in blue and humpback whale BIAs highlight areas of high risk as well as regions with relatively little strike hazard. For those areas with negligible strike risk (San Nicholas Island and Monterey Bay to Pescadero Point for blue whales, Point Arena to Fort Bragg for both blue and humpbacks, and Stonewall and Heceta Bank and Point Saint George for humpback whales), ensuring that changes in vessel traffic do not compromise the ecological refuges is a vital part of a successful conservation strategy. Alternately, Santa Barbara Channel to San Miguel BIAs are high threat areas for both blue and humpback whales and deserve high priority for protection and regulation. For humpback whales, regulation in the Gulf of the Farallones-Monterey Bay BIA is high priority with the potential to mitigate 17% of total mortality. Designation of these ecologically important areas as Marine Protected Areas (MPAs) or Seasonal Management Areas (SMAs) could enable regulation and management to protect whales as has been done for right whales on the east coast [7,71].

Differences in mortality distribution for the three species results in different priority areas for strike mitigation. Ship strikes are most important for humpbacks off the Bay Area and in the Greater Farallones NMS, while the most strike risk for blue whales is in the Southern California Bight. The more offshore distribution of fin whales (Fig 1) may present challenges in balancing mitigation strategies with the other two species since shifting vessel traffic offshore may elevate fin whale strike risk. While more specific priority areas exist for each species, the 10% of area with highest mortality coincides well for all three stocks (Fig 4). This area, defined in our analysis as the region with mortality greater than the 90^th^ percentile, serves as a priority location for broader-scale mitigation efforts such as slow-steaming regulations. Putting in place regulations that cover the 90^th^ percentile areas can mitigate the majority of mortality for all three species.

Vessel strikes offshore of California have been intimately related to progressive vessel air pollution regulations over the last decade. In 2009, the California Air Resources Board (CARB) instituted limits on the sulfur content of fuel burned by ships within 24 Nm (44.5 km) of the coast [39] and tightened the limit in 2014 [18]. As a result, to save on the higher cost of low-sulfur fuel, vessels shifted travel to routes that reached the 24 Nm limit more directly and skirted the edge of the regulatory zone. These changes inadvertently altered the vessel overlap with whale populations [18]. In 2015, the Environmental Protection Agency (EPA) instituted regulations for the entire 200 Nm EEZ that were similar to the CARB rule. While the results of this change are yet unknown, vessels may shift their passage back inshore, likely increasing overlap with blue and especially humpback whales. It is also important to note that our results do not consider potential future changes in whale distribution or population levels. Current information suggests that blue whales may shift their feeding distribution in the eastern North Pacific in response to ocean/prey conditions [27], while the fin whale population is increasing rapidly [30]. These changes and possible shifts in distribution resulting from climate change [72] will likely alter strike mortality and should be considered in planning of strike management. The density habitat model [40] we used for spatial density of whales in our model includes dynamic oceanographic variables that could not be incorporated here. Future efforts could take advantage of these ecological relationships to further inform management.

The model we developed relies on a number assumptions and uncertainties but is useful for identifying key areas where research is needed to help evaluate ship strike risk over broad regions. How whales react to or avoid approaching ships and how this varies with vessel speed is a key parameter that made significant differences in our estimates depending on which of three scenarios we used. Additionally, there are likely differences in this parameter among the three species we included given their variation in body size, morphology, maneuverability, and social behavior. These differences could alter their relative risk of ship strike. In addition, the effect of vessel draft on probability of collision is a second key parameter that needs further investigation. To date, only limited trials of scale models have provided information on strike zone depth [44].

The spatial model we used [40] is based on fairly course scale surveys that cover primarily offshore waters, so do not capture the finer scale density patterns in the nearshore waters where most of the shipping lanes are. Finer scale data and models have been developed for some species in some of these key areas [21], but would need to be expanded to be applicable to the broader area we examined. Finally, better information is needed on occurrence of ship strikes both through better reporting of strikes and development of better species-specific carcass recovery rates to quantify the proportion of mortality that is documented through strandings.

Achieving empirical quantification of strike mortality is likely unachievable, so validation of our model results presents a challenge. Some current efforts and potential research may provide further insight. Work is underway to further validate and improve our estimates of vessel avoidance and strengthen one of the more uncertain parameters in our model. Concurrent vessel position data and whale tracking can also provide enhanced insight into the likelihood and prevalence of encounters and strikes (e.g. [12]). To enhance independent estimates of strike frequency, automated detection of beached whale carcasses currently under development could minimize unobserved strike events. Methods to detect strike events using vessel engine monitoring combined with AIS data are also being explored. Finally, exploration of observed population trends using population models and conservative species vital rates has both the potential to check the viability of our estimates and to provide valuable context regarding the importance of strike management strategies to the growth and viability of these populations.

To achieve successful reduction of ship strike deaths of whales on the U.S. West Coast, we recommend four important strategies: 1) further efforts to re-locate shipping lanes away from high density areas of whales; 2) extension of lanes further offshore so that high-traffic routes between ports are shifted away from coastal concentrations of whales; 3) creation of ‘Areas to be Avoided’ in cooperation with the International Maritime Organization such as those surrounding the northern Channel Islands and in the waters off Washington’s Olympic Coast; and 4) implementation of a graduated slow-steaming requirement within the U.S. Exclusive Economic Zone where ships travel at increasingly reduced speed as they travel closer to shore. This fourth recommendation is the most extreme and likely most controversial but also has the greatest potential to mitigate the widespread threat of vessel strikes. In addition, broad-scale speed reduction has the added benefit of decreasing pollution [73], carbon emissions and fuel costs, at least partially offsetting the price to the shipping industry of longer transit times. Since broad spatial mitigation of strikes appears to be necessary for substantial strike reduction, a priority area of research should be predicting the economic impacts and identifying opportunities for economic incentives such as carbon payments that could offset costs of a slow-steaming regulation. We also stress the importance of continued and enhanced conservation measures in the TSSs given their high risk intensity and established regulatory framework. Combining these measures in the regions with highest risk represents a coordinated strategy with real potential to decrease ship strike mortality significantly.

## Supporting information

S1 File Time-at-depth for blue, humpback and fin whales. (XLSX)

S2 File Testing the effects of parameter uncertainty. (DOCX)
