# Correction: High mortality of blue, humpback and fin whales from modeling of vessel collisions on the U.S. West Coast suggests population impacts and insufficient protection

**DOI:** 10.1371/journal.pone.0201080

**Published:** 2018-07-16

**Authors:** R. Cotton Rockwood, John Calambokidis, Jaime Jahncke

The column heading "Mortality per 10^4^ km^2^" for Tables 4–9 appears incorrectly. Please see the corrected Tables 4–9 here.

**Table 4:** Statistics of blue whale strike mortality within the Biologically Important Areas (BIAs) defined in Calambokidis et al. 2015. Total mortality, mortality per 100,000 km^2^ and percent of total study area mortality are reported for each BIA.

Name	BIA Area (km^2^)	Months of peak abundance	Area Mortality	Mortality per 10^5^ km^2^	Percent of total mortality
Gulf of the Farallones	5,243	July—November	<1	14	4%
Monterey Bay to Pescadero Pt	2,378	July—October	<1	4	<1%
Santa Barbara Channel to San Miguel	1,981	June—October	<1	22	2%
Pt Conception to Pt Sal	1,743	June—October	<1	9	<1%
Pt Arena to Fort Bragg	1,419	August—November	<1	1	<1%
Santa Monica Bay to Long Beach	1,187	June—October	<1	51	3%
Tanner-Cortez Bank	1,076	June—October	<1	5	<1%
San Diego	984	June—October	<1	20	1%
San Nicholas Is	427	June—October	<1	3	<1%

**Table 5:** Statistics of humpback whale strike mortality within the Biologically Important Areas (BIAs) defined in Calambokidis et al. 2015. Total mortality, mortality per 100,000 km^2^ and percent of total study area mortality are reported for each BIA.

Name	BIA Area (km^2^)	Months of peak abundance	Area Mortality	Mortality per 10^5^ km^2^	Percent of total mortality
Gulf of the Farallones—Monterey Bay	9,761	July—November	3	32	17%
Northern WA	3,393	May—November	<1	6	1%
Stonewall and Heceta Bank	2,573	May—November	<1	<1	<1%
Santa Barbara Channel—San Miguel	2,639	March—September	<1	15	2%
Morro Bay to Pt Sal	1,908	April—November	<1	2	<1%
Fort Bragg to Pt. Arena	1,591	July—November	<1	2	<1%
Pt St. George	1,233	July—November	<1	<1	<1%

**Table 6:** Mortality statistics for shipping lanes. Mortality per 100,000 km^2^ and percent of total study area mortality are also reported for each region.

		Blue Whales			Humpback Whales			Fin Whales		
Region	Area (km^2^)	*Area Mortality*	*Mortality per 10*^*5*^ *km*^*2*^	*Percent of total mortality*	*Area Mortality*	*Mortality per 10*^*5*^ *km*^*2*^	*Percent of total mortality*	*Area Mortality*	*Mortality per 10*^*5*^ *km*^*2*^	*Percent of total mortality*
San Francisco	1,136	1	65	4%	3	255	13%	<1	20	<1%
Southern California	2,314	1	61	8%	<1	36	4%	1	48	3%
Washington	166	<1	4	<1%	<1	6	<1%	<1	<1	<1%
Waters outside lanes	798,015	16	2	86%	18	2	81%	41	5	96%

**Table 7:** Mortality within National Marine Sanctuaries. Mortality per 100,000 km^2^ and percent of total study area mortality are also reported for each sanctuary.

		Blue Whales			Humpback Whales			Fin Whales		
National Marine Sanctuary	Area (km^2^)	*Area Mortality*	*Mortality per 10*^*5*^ *km*^*2*^	*Percent of total mortality*	*Area Mortality*	*Mortality per 10*^*5*^ *km*^*2*^	*Percent of total mortality*	*Area Mortality*	*Mortality per 10*^*5*^ *km*^*2*^	*Percent of total mortality*
Cordell Bank	3,331	<1	12	2%	1	34	5%	<1	10	<1%
Channel Islands	3,818	<1	13	3%	<1	6	1%	<1	10	<1%
Greater Farallones	8,548	1	14	7%	4	49	19%	<1	7	1%
Monterey Bay	15,795	<1	5	4%	3	18	13%	2	10	4%
Olympic Coast	8,259	<1	<1	<1%	<1	9	4%	<1	<1	<1%

**Table 8:** Mortality within sovereign waters (3 Nm offshore) for each of the three west coast states, California, Oregon and Washington. Mortality per 100,000 km^2^ and percent of total study area mortality are also reported for each state.

		Blue Whale			Humpback Whale			Fin Whale		
Jurisdiction Name	Area (km^2^)	*Area mortality*	*Mortality per 10*^*5*^ *km*^*2*^	*Percent of total mortality*	*Area mortality*	*Mortality per 10*^*5*^ *km*^*2*^	*Percent of total mortality*	*Area mortality*	*Mortality per 10*^*5*^ *km*^*2*^	*Percent of total mortality*
California	14,280	<1	5	4%	<1	4	3%	<1	2	<1%
Oregon	3,268	<1	<1	<1%	<1	2	<1%	<1	<1	<1%
Washington	2,049	<1	<1	<1%	<1	2	<1%	<1	<1	<1%

**Table 9:** Mortality on and off the continental shelf (defined by the 200-meter isobath). Mortality per 100,000 km2 and percent of total study area mortality are also reported for each region.

		BLWH Mortality			HUWH Mortality			FIWH Mortality		
Area Name	Area (km^2^)	*Area mortality*	*Mortality per 10*^*5*^ *km*^*2*^	*Percent of total mortality*	*Area mortality*	*Mortality per 10*^*5*^ *km*^*2*^	*Percent of total mortality*	*Area mortality*	*Mortality per 10*^*5*^ *km*^*2*^	*Percent of total mortality*
Continental shelf	57,030	3	5	15%	8	14	36%	1	2	3%
>200m	754,905	15	2	85%	14	2	64%	41	6	97%

In the Results and Discussion sections, the units for "mortality per area" and "strike intensity" also appear incorrectly. The correct units for "mortality per area" and "strike intensity" should be 10^5^ km^2^.
