# Supplementary material for: High mortality of blue, humpback and fin whales from modeling of vessel collisions on the U.S. West Coast suggests population impacts and insufficient protection
Source: PLoS One. 2017 Aug 21;12(8):e0183052. doi: 10.1371/journal.pone.0183052 (PMC5565115; doi:10.1371/journal.pone.0183052)
Supplement: S2 File — (DOCX) [file pone.0183052.s002.docx]

# S2 File. Testing the effects of parameter uncertainty.

## Strike zone definition

Since whales and ships co-habit a three-dimensional medium, depth is an important determinant of potential for collisions. As air-breathing mammals, whales are tied to the surface, yet they spend significant time diving and foraging at depth in the water column. Vessels utilize the top of the water column to a depth which varies by individual vessel as well as vessel load (also called ‘draft’ – the distance from the water line to the bottom of a ship’s hull). In addition, experiments with scale models of ships and whales determined that hydrodynamic forces from the passage of the vessel can result in a collision even when the animal is up to twice the vessel draft in depth (Silber, Slutsky, & Bettridge, 2010). This research performed a limited number of trials and defined a ‘risk zone’, but also showed that the likelihood of a strike varies within that zone. When the whale model was placed directly in the path of the vessel at the surface, 100% of trials resulted in collisions. When the whale was at the ship depth or twice the draft depth, however, the collision rate dropped to 50%.

To maintain a conservative approach in our main models, we chose to use the draft depth as the risk zone. Since evidence suggests that collisions may occur at up to twice the vessel draft or more, here we test the effect of using 1.5x and 2x the vessel draft as the strike depth. Mortality using 1.5x the draft increased by 4.92, 2.26 and 8.56 deaths for blue, humpback and fin whales, respectively (Table A). That equates to changes relative to our main model of 27.4%, 10.4% and 20.0%. For a 2x vessel draft strike zone, mortality increased by 8.21 (45.6% increase), 3.79 (17.4% increase) and 15.85 (37.1% increase) deaths for blue, humpback and fin whales, respectively. Given that only 50% of trials at 2x draft were strikes, using this depth is likely to result in an overestimate of mortality since the model formulation inherently assumes 100% of encounters within the strike zone will lead to strikes (excluding avoidance behavior). Nonetheless, this test shows that the definition of the strike zone, as well as how collision risk changes continuously within that zone, are important parameters. The results highlight the importance of further research to refine the strike zone parameter. Further model tests as well as computer simulations are the most promising approaches given the inherent difficulty of real-life experimentation.

## Whale swim speed

Whales exhibit a significant range of swim speeds from resting to sprinting that can be invoked as an escape or avoidance response (Ford & Reeves, 2008). Since encounter rate depends on the speed of both vessels and whales, we tested the effect of plausible mean swimming speeds associated with three behavior repertoires: area restricted search, travel and all behaviors averaged. Area restricted search is associated with slower swim speeds and higher turn angles observed during foraging and other geographically restricted activities. Travel is linked to long-distance migration or movement between preferred feeding locations.

We searched the literature to find swimming speeds quantified using electronic tags for each of the study species and classified under each behavior. Swim speed was calculated as the mean of all records found (Table B). Where values were reported as the mean of multiple individuals, the mean speed was weighted by the number of samples (individual whales) used in the calculation. Mortality estimates between swim behaviors changed as little as 0.11 deaths and as much as 1.24 deaths (Table C). These differences equated to 0.24% change and 1.29% respectively. Though still small, the largest difference in prediction occurred for fin whale traveling behavior. Given the small effect of swim speed parameterization on mortality predictions, we conclude that further research on swim speeds, including variation across space, should not be a priority for quantifying strike risk and mortality.

## Avoidance Model 1

Avoidance Model 1 relies on several studies that have found a negative and non-linear relationship between ship speed and successful avoidance behavior by whales. In particular, Gende (2011) found a difference in average closest point of encounter between whales and cruise ships of 114 m above and below vessel speeds of 11.8 knots (6.1 m/s). We interpret the difference in closest distance as a sign of delayed reaction to faster oncoming vessels. We therefore, defined an inflection point for our model at 11.8 knots. We also expect that as vessel speeds increase, the ability of whales to avoid collision will approach zero. Using a representative large-vessel draft of 15 m, a hypothetical response distance of 1,200 m (observed for some whales in McKenna et al. (2015)) and dive rates of 0.6 to 1.0 m/s (mean and maximum descent of ‘avoidance dives’ (McKenna et al., 2015)), speeds that would make avoidance unlikely range from 48-80 m/s. We therefore parameterized our curve to have <10% avoidance probability at 48 m/s. Using these parameters, we defined Model 1 as

$$P\left( Avoidance \right)= \frac{0.9}{1+e^{-0.15{( v}_{b}-6.1)}}$$

Where $v_{b}$ is the vessel velocity and 6.1 is 11.8 knots converted to m/s (Fig A).

## Figures and Tables

**Table A. Comparison of mortality estimates depending on the definition of the strike zone as 1, 1.5 or 2 times the vessel draft.** Absolute and percent differences are referenced to 1x mortality.

|  | **1x Vessel Draft Mortality** |  | **1.5x Vessel Draft** | | |  | **2x Vessel Draft** | | |
| --- | --- | --- | --- | --- | --- | --- | --- | --- | --- |
| **Species** |  |  | Mortality | Absolute difference | Percent difference |  | Mortality | Absolute difference | Percent difference |
| Blue whale | 17.98 |  | 22.91 | 4.92 | 27.4% |  | 26.19 | 8.21 | 45.6% |
| Humpback whale | 21.78 |  | 24.04 | 2.26 | 10.4% |  | 25.57 | 3.79 | 17.4% |
| Fin whale | 42.73 |  | 51.29 | 8.56 | 20.0% |  | 58.58 | 15.85 | 37.1% |

**Table B. Swim speeds derived from published data gathered with GPS tracking tags.**

| **Species** | **Behavior** | **Speed (km/hr) (Mean ± SD)** | **n** | **Source(s)** |
| --- | --- | --- | --- | --- |
| Blue Whale | Mean | 2.64 ± 1.23 | 92 | Bailey et al. 2009 |
|  | Traveling | 3.70 ± 2.15 | 92 | Bailey et al. 2009 |
|  | ARS | 1.05 ± 0.97 | 92 | Bailey et al. 2009 |
| Humpback whale | Mean | 3.37 ± 1.30 | 16 | Mate et al. 1989; Lagerquist et al. 2008 |
|  | Traveling | 4.20 ± 1.00 | 6 | Mate et al. 1989; Lagerquist et al. 2009 |
|  | ARS | 2.56 ± 0.75 | 4 | Mate et al. 1989; Lagerquist et al. 2010 |
| Fin Whale | Mean | 3.48 ± 1.22 | 16 | Schorr et al. 2010; Silva et al. 2013 |
|  | Traveling | 6.37 ± 1.97 | 18 | Schorr et al. 2010; Silva et al. 2014 |
|  | ARS | 2.55 ± 1.31 | 12 | Schorr et al. 2010; Silva et al. 2015 |

**Table C. Difference in mortality for all models predicted based on swim speeds associated with ARS and traveling behavior as compared to mean speed across all behaviors.**

|  |  | **Mean Swim Speed** Mortality |  | **ARS Swim Speed** | | |  | **Traveling Swim Speed** | | |
| --- | --- | --- | --- | --- | --- | --- | --- | --- | --- | --- |
| **species** | **model** |  |  | Mortality | Absolute Difference | Percent Difference |  | Mortality | Absolute Difference | Percent Difference |
| BLWH | 1 | 42.34 |  | 42.19 | 0.15 | 0.35% |  | 42.51 | 0.17 | 0.41% |
|  | 2 | 19.05 |  | 18.98 | 0.07 | 0.35% |  | 19.13 | 0.08 | 0.41% |
|  | 3 | 19.48 |  | 19.42 | 0.06 | 0.33% |  | 19.56 | 0.07 | 0.38% |
| HUWH | 1 | 52.24 |  | 52.04 | 0.19 | 0.37% |  | 52.49 | 0.25 | 0.48% |
|  | 2 | 23.51 |  | 23.42 | 0.09 | 0.37% |  | 23.62 | 0.11 | 0.48% |
|  | 3 | 23.22 |  | 23.14 | 0.08 | 0.33% |  | 23.32 | 0.10 | 0.44% |
| FIWH | 1 | 96.26 |  | 96.01 | 0.24 | 0.25% |  | 97.50 | 1.24 | 1.29% |
|  | 2 | 43.32 |  | 43.21 | 0.11 | 0.25% |  | 43.87 | 0.56 | 1.29% |
|  | 3 | 46.31 |  | 46.20 | 0.11 | 0.24% |  | 46.88 | 0.57 | 1.23% |


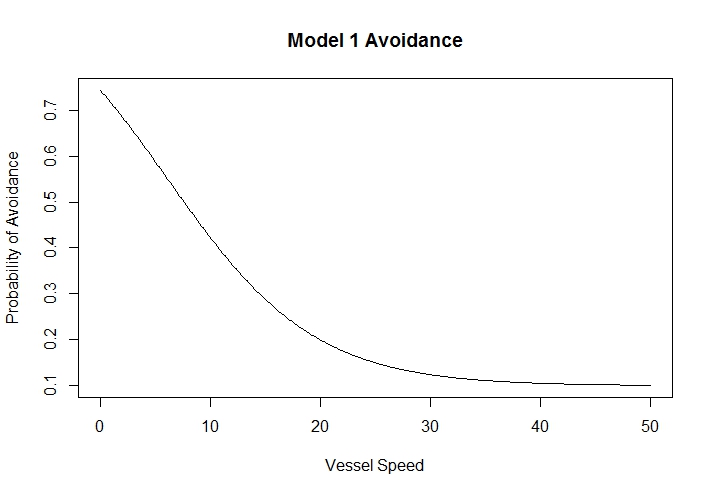


**Fig A. Model 1 relationship between vessel speed and probability of behavioral avoidance of collision by a whale.**
